# Investigating the Prevalence of Sleep Disorder and the Impact of Sweet Almond on the Quality of Sleep in Students of Tehran, Iran

**Published:** 2019-06

**Authors:** Jafar GHAFARZADEH, Khosro SADEGHNIIAT-HAGHIGHI, Omid SADEGHPOUR, Samaneh AKBARPOUR, Farshad AMINI-BEHBAHANI

**Affiliations:** 1. Research Institute for Islamic and Complementary Medicine, Iran University of Medical Sciences, Tehran, Iran; 2. School of Persian Medicine, Iran University of Medical Sciences, Tehran, Iran; 3. Occupational Sleep Research Center, Baharloo Hospital, Tehran University of Medical Sciences, Tehran, Iran

**Keywords:** Insomnia, Insomnia severity index, Sweet almond

## Abstract

**Background:**

Insomnia is an important problem in medical sciences students and has implications for their educational progress. The current study aimed to estimate the prevalence of sleep disorders and investigating the impact of sweet almond on quality of sleep in students of the Tehran University of Medical Sciences (TUMS), Tehran, Iran who live in dormitories.

**Methods:**

This is a before-after study conducted in 2017. At first, using the ISI questionnaire prevalence of sleep disorders was determined. Sweet almond was the study intervention. Each day, 10 almonds were given to 446 students for 14 d. At the end of the second week, again ISI questionnaire was filled. SPSS was used to analyze data. The McNemar, Wilcoxson Signed Ranks, and Repeated Measures tests were used.

**Results:**

Out of 442 participants, 217 (49.1%) were female. Before intervention, 343 (77.6%) had insomnia and 99 (22.4%) had normal sleep. After intervention, 306 (69.2%) had insomnia and 136 (30.8%) had normal sleep. Having sweet almond for two weeks is associated with reducing insomnia (*P*<0.05). Investigating the almond impact in different categories also showed that it has a reducing impact on severe, mild, weak and normal sleep categories (*P*<0.05).

**Conclusion:**

Sweet almond has impacts on quality of sleep of those students of the TUMS that are living in dormitories. Intervention programs to improve quality of sleep are necessary and with regard to the high prevalence of insomnia, students must be protected, guided and consulted.

## Introduction

The insomnia prevalence in normal populations is from 15% to 42%, and its prevalence among university students is from 19.17% to 57.5% (1–7). Medical University students, because of higher-education stresses, have higher levels of insomnia (8–13). Most of students to have somehow sleep disorders. In a study on the student’s quality of sleep found that 31% of participants have weak or too weak quality of sleep (14). The student’s quality of sleep was investigated and found that disorder in falling asleep, morning fatigue, daily sleepiness, nightmares and wake up earlier than usual in the morning have a high prevalence among students (15). Students of medical universities, because of the need for night works (e.g. night shifts in hospitals), high stress and work pressure, usually have sleep disorders. Moreover, living in a dormitory may decrease the quality of sleep. In another study, medical students had higher levels of sleep disorders, daily sleepiness or sleepiness during classes (14). 5.75% of students were sleepiness during the day (16). Individuals with sleep disorders usually complain of daily sleepiness, memory impairment, problems in concentration and thinking, restlessness and irritability, intolerance to stress, and performing abstract and complex tasks, and it is associated with undesirable quality of sleep, poor performance, and dissatisfaction from educational progress (9, 12).

Prevalence of insomnia is 2 to 5 times higher in females than males (17). In a study on sleep and awakening patterns of students, 69.7% of students with poor educational performance had problems with sleeping and 65.6% reported that because of sleep disturbances continuously wake during nights, and 72.7% reported that due to the low quality of sleep have problems with concentration and attention (18).

Few studies have investigated the impact of the sweet almond on quality of sleep. The sleeping effects of sweet almond were showed on rats (19). Additionally, this issue is mentioned in traditional medicine texts of Iran (20). However, adequate attention is not paid to it.

The current study aimed to investigate the prevalence of insomnia and impacts of sweet almond on quality of sleep in dormitories resident students of the Tehran University of Medical Sciences (TUMS).

## Methods

This cross-sectional study used a before-after design to compare the student’s quality of sleep in 2017. At first, the quality of sleep was calculated using the ISI questionnaire.

Sweet almond was considered as an intervention. Then, for two weeks, 10 almonds were given to 446 students who were living in dormitories. At the end of the second week, again the students’ quality of sleep was calculated using the ISI questionnaire. Due to the short duration of the study, participants were both case and control. The validity of the Persian version of the questionnaire is confirmed by the Sleep Disorders Research Center of the TUMS. The ISI questionnaire comprises of 5 main questions, which its first question is divided into 3, and has a Likert scale scoring (never, weak, average, severe, too severe). It uses the following scoring system:

- The Insomnia Severity Index
- Guidelines for Scoring/Interpretation:
  Add scores for all seven items (1a+ 1b+ 1c+ 2+3+4+5)
- Total score ranges from 0 to 28
- 0–7 = No clinically significant insomnia
- 8–14 = Subthreshold Insomnia
- 15–21 = clinical Insomnia (moderate severity)
- 22–28 = Clinical Insomnia (severe)

Those students of the TUMS who were living in dormitories were considered as the study population. Inclusion criterion was being at least the second-year student. Exclusion criteria were as follows: Interns and Residents, and students with a history of psychological disorders. To determine the sample size, there is no previous study on the impacts of the sweet almond on humans, a study on the impacts of sweet almond on rats (19) was used, which based on this study the *P* was considered as equal to 0.3. The following formula was used to determine the sample size: N=z(1-a/2) p(1-p)/d2

By using the following values, the sample size was calculated as equal to 442.

- *P*=0.30
- D=0.03
- N= (1.96) (0.975) (0.30) (0.7)/0.0009
- a=0.05
- z(1-a) =1.96
- n=446

The TUMS has many dormitories, three dormitories were selected, by considering participants gender, then 446 questionnaires were randomly given to students. Students’ participation was voluntary and their consent was the first priority. The study protocol was approved by the Ethical Committee of the Iran University of Medical Sciences (ethical code: IR.IUMS.REC.1395.9321309005).

The SPSS software version 24(Chicago, IL, USA) was used for data analysis. The McNemar, Wilcoxson Signed Ranks, and Repeated Measures tests were used.

## Results

Overall, 446 questionnaires were distributed and 442 participated in study. The response rate was 99.1%. Among 442 participants, 217 (4931%) were female and 225 (50.9%) male. Questionnaires were filled two times (before and after the intervention). At the first time, required information to calculate the quality of sleep were collected. Then, to calculate the insomnia score, initially, participants based on their sleep score were divided into two groups: normal sleep (sleep score from 1 to 7), and insomnia (sleep score higher than 7).

In the previous stage, 343 (77.6%) students had insomnia, that 248 (56.1%) were mild, 90 (20.4%) moderate, and 5 (1.1%) severe. 99 students (22.4%) had normal sleep. In after stage, 306 (69.2%) students had insomnia, that 245 (55.4%) were mild, 58 (13.1%) moderate, and 3 (0.7%) severe, and 136 (30.8%) had normal sleep (Chi-Square=10.891 P<0.001). The McNemar’s test was employed to investigate the impact of sweet almond on insomnia. It has a positive impact on reducing insomnia (*P*<0.05). The Wilcoxon Signed Ranked test was used to investigate the impact of sweet almond on the above-mentioned groups. The results revealed that it had a significant impact on reducing insomnia (Table 1).

**Table 1:** Sweet almond’s impact on each insomnia group

**Variable**		**Number**	**Rabke average**	**Sum of ranks**
Insomnia level after having sweet almond	Negative ranks	135 ^a^	103.56	13980.00
Insomnia level before having sweet almond	Negative ranks	69 ^b^	100.43	6930.00
	Ties	238 ^c^		
Z	^d^ −4.679			
Two-side significance	<0.001			

a The insomnia level before having the sweet almond> insomnia level after having the sweet almond

b Insomnia level after having the sweet almond> insomnia level before having the sweet almond

c Insomnia level after having the sweet almond= insomnia level before having the sweet almond

d Based on the positive ranks

**Fig. 1:**
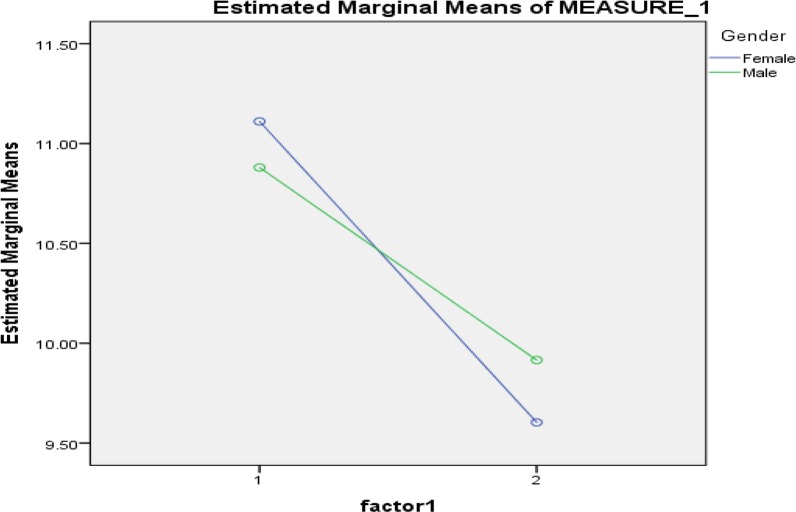
Sweet almond’s impact on insomnia

Finally, to examine the impact of gender on insomnia score before and after the intervention, the repeated measures test was employed. The results showed gender had not any impact on the association between having sweet almond and insomnia (*P*=0.912) (Fig. 1).

## Discussion

The current study aimed to investigate the prevalence of insomnia and the impact of sweet almond in students of the TUMS who living in dormitories. There are various methods to improve students’ quality of sleep and good improvements have been made in this field (21).

In the current study, the prevalence of insomnia before and after the intervention was 77 and 69%, respectively. Many of the cases where having mild insomnia. Only 14% of participants are satisfied with their night sleep (22). 40.6% of students were not satisfied from their sleep (23). In a study on quality of sleep, medical students did not have a good quality of sleep, and female students have higher levels of insomnia (24). Students have good quality of sleep (15). Since the population study of the current study is students who are studying various fields of medical sciences and living in dormitories, those studies which to somehow are consistent with our study can be used in the interpretation of the results. Most of the students, with regard to the dormitories contexts and the nature of their study field, are suffering from insomnia and are in need of various interventions. In the current study, intervention was the sweet almond, each day 10 almonds for two weeks. Overall, 442 students from different fields of medical sciences accepted to participate. The findings indicate that sweet almond reduces insomnia (*P*<0.001). Different studies examined the impact of sweet almond on insomnia and its related problems. In a study on traditional medicine of Iran, the ancient books related to medicine were reviewed and found 25 herbal substances which reduce insomnia, including sweet almonds. They discussed sedative and sleepiness effects of it (20). Animal studies also have proven sedative impacts of sweet almonds. Sweet almonds impacts were investigated on rats. Overall, 400 mg sweet almond had sedative impacts on rats (19). The findings of the current study also showed that sweet almond is associated with significant reduction in insomnia that is consistent with another. The impact of the Aroma of Lavandula Angustifolia and almond oil was examined on reducing insomnia. Both of them significantly reduce insomnia, but the latter had lesser impacts (25). A few studies with a large scale population have investigated the impact of sweet almond on insomnia. Then, the current study can be helpful in diminishing limitations and comparing findings.

One of the main limitations of the current study is different fields of students and their study hours, with various impacts on insomnia. Since each student is compared to her or himself, it is to somehow address. The study is conducted at the beginning of the semester, before starting exams that create stress.

## Conclusion

Sweet almond had impacts on quality of sleep. These findings can expand the knowledge on examined variables; as well can be used in future research, even in other groups. Practically, with regard to the significant association between sweet almond and sleep, it reveals the need for interventions to improve the quality of sleep. Moreover, based on the results on the prevalence of insomnia, students must be protected, guided and consulted, and required measures to improve their quality of sleep be performed. Future research, examine the impact of sweet almond along with other psychological factors and among other age groups, different education levels, and students of other students based on the demographic factors.

## Ethical considerations

Ethical issues (Including plagiarism, informed consent, misconduct, data fabrication and/or falsification, double publication and/or submission, redundancy, etc.) have been completely observed by the authors.
